# Illusional Perspective across Humans and Bees

**DOI:** 10.3390/vision6020028

**Published:** 2022-05-31

**Authors:** Elia Gatto, Olli J. Loukola, Maria Elena Miletto Petrazzini, Christian Agrillo, Simone Cutini

**Affiliations:** 1 Department of Chemical, Pharmaceutical and Agricultural Sciences, University of Ferrara, 44121 Ferrara, Italy; 2 Department of Life Sciences and Biotechnology, University of Ferrara, 44121 Ferrara, Italy; 3 Ecology and Genetics Research Unit, University of Oulu, P.O. Box 3000, FI-90014 Oulu, Finland; olli.loukola@oulu.fi; 4 Department of General Psychology, University of Padova, 35131 Padova, Italy; mariaelena.milettopetrazzini@unipd.it (M.E.M.P.); christian.agrillo@unipd.it (C.A.); 5 Department of Developmental and Social Psychology, University of Padova, 35131 Padova, Italy; simone.cutini@unipd.it; 6 Padua Neuroscience Center, University of Padova, 35129 Padova, Italy

**Keywords:** bees, invertebrates, visual illusion, visual perception

## Abstract

For two centuries, visual illusions have attracted the attention of neurobiologists and comparative psychologists, given the possibility of investigating the complexity of perceptual mechanisms by using relatively simple patterns. Animal models, such as primates, birds, and fish, have played a crucial role in understanding the physiological circuits involved in the susceptibility of visual illusions. However, the comprehension of such mechanisms is still a matter of debate. Despite their different neural architectures, recent studies have shown that some arthropods, primarily Hymenoptera and Diptera, experience illusions similar to those humans do, suggesting that perceptual mechanisms are evolutionarily conserved among species. Here, we review the current state of illusory perception in bees. First, we introduce bees’ visual system and speculate which areas might make them susceptible to illusory scenes. Second, we review the current state of knowledge on misperception in bees (Apidae), focusing on the visual stimuli used in the literature. Finally, we discuss important aspects to be considered before claiming that a species shows higher cognitive ability while equally supporting alternative hypotheses. This growing evidence provides insights into the evolutionary origin of visual mechanisms across species.

## 1. Introduction

“I’m havin’ illusions, all this confusion’s drivin’ me mad inside”—Cypress Hill.

What we define as “reality” arises from the integration of multiple sensory stimuli that individuals experience in their lives. The perceptual system, therefore, provides us with a limited representation of reality, which comprises only what we can perceive. Indeed, our sensory system does not allow us to perceive some aspects that define the environment, such as radio waves, non-aromatic chemical compounds, and microelements such as chromatin and bacteria [1].

However, the human mind can create a representation of the external world, albeit limited to what we can perceive and to which we are able to respond. This system seems efficient in a static situation, in which conditions are limited and allow an individual to predict their outcome easily. However, realistic environments are dynamic, and individuals must quickly adapt to unpredictable situations. Natural selection should favor those individuals that respond flexibly to changing environments based on their experiences. Therefore, our perceptual system allows us to characterize a situation by searching for common principles. For example, in the visual system, visual sensory input is acquired from the retina and transmitted by the brain to generate a perceptual image. It is interesting to note that some phenomena generate conflicts between what we perceive and the conception of what we consider reality, which we have created by our experience. These phenomena are defined as illusions [1].

Among the illusory phenomena, visual illusions have been widely investigated to help us understand how sensory information is processed. These classes of illusions are often caused by the spatial conformation of the elements present in the scene, which generates a conflict between what our mind processes and what the physical object really is. The main information that triggers these illusions is visual, but the works of Shams and collaborators on the induction of visual illusions when we perceive sound stimuli are intriguing [2]. According to Gregory [3], we can classify illusions into two categories according to their source. “Physical illusions” refer to phenomena generated by the malfunction between a physical phenomenon and the information the retina acquires, such as the refraction of light underwater or images seen in a mirror. The second category, of the greatest interest, concerns “cognitive illusions”, which are generated by the conflict between visual information our perceptual system acquires and the brain’s consequent processing. Phenomena such as the Ebbinghaus, Müller-Lyer, and Ponzo illusions and the Kanizsa triangle are cognitive illusions [3]. These illusions are created by the false perception that an image generates in our mind. These phenomena are caused by our perceptual visual system. Indeed, the system converts the two-dimensional visual information the retina acquires into a three-dimensional representation with an image with which we have experience. Our system, therefore, has adapted a set of transformation rules to provide fast answers to what we perceive. Consequently, the interpolation of these rules can be misleading when the information generates a conflict with what we expect.

Animals live in a dynamic environment that comprises an enormous amount of sensory information, and their nervous systems need to acquire, process, and integrate the most relevant information to make adaptive decisions. From an evolutionary point of view, different species might evolve similar rule-based strategies which are generally applied when perceiving similar visual inputs [4]. Therefore, we should not be surprised that other species are susceptible to optical illusions similar to those humans experience.

Previous reviews have thoroughly discussed the current knowledge of several illusory phenomena among vertebrates [5,6,7,8]. However, limited knowledge is available for invertebrate species. Vertebrates and invertebrates have divergently evolved from a common predecessor for at least 600 million years; the latter are characterized by a relatively simple sensory system adapted for faster reaction [9]. However, similarities in physiological and morphological systems have been found to be highly conserved among species, raising the question of the evolutionary origin of these systems [10,11,12]. An assessment of animal illusion susceptibility can reveal whether and how environmental and evolutionary factors have affected our perceptual systems.

This review focuses on the visual perception of invertebrates, in particular bees. The impressive cognitive capacities of bees, including the formation of abstract concepts such as sameness and difference [13], numerosity [14], and holistic processing of the human face [15], have aroused interest in understanding the visual pathways in a relatively small brain. First, we describe the visual system of bees and speculate which areas might be involved in susceptibility to illusory figures. Second, we review the current literature on misperception in bees (Apidae) by focusing on the established methodologies and illusory visual stimuli used in the literature. Finally, we discuss important aspects that authors should take into consideration before claiming that a species shows cognitive similarities with “higher species”, such as humans, when alternative “low-level” explanations are equally possible.

## 2. Bee Perspective

Freely moving organisms are in constant interaction with the environment. The nature of stimuli that an animal perceives influences its behavior. It is worth noting that animals, on the one hand, can acquire visual information through single-aperture or compound eyes [16]. The former is characterized by a retina-like structure, that is, a non-uniform distribution of photoreceptor cells, which improves sensitivity and resolution. Invertebrates, on the other hand, have a variety of eye structures, from simple photoreceptors to complex structures such as a compound eye. Unlike single-aperture eyes, compound eyes consist of thousands of single image systems (ommatidia) arranged spherically on the surface of the eye. Each unit can collect partial images of the target object, and then the entire image is recreated from the separately acquired information. As a result of this organization, the compound eye has poor spatial resolution but a wider field of view that permits better detection of moving objects. To increase image resolution, invertebrates need to spend more time scanning information before making a decision. For example, many invertebrates actively sample the scene from a close distance, scanning the pieces that constitute the scene one-by-one, and the scanning time is correlated with the complexity of images [17,18]. Indeed, bees often need longer scanning times to acquire the necessary information to make a decision. Such behavior, known as “active vision”, compensates for the absence of simultaneous processing of an entire image at a glance [19]. Honeybees (*Apis mellifera*) have been shown to process visual information in a different manner and use a different strategy than humans do in a visual discrimination task that uses stimuli commonly found in numerical cognition studies [20]. Bees used continuous cues such as edge length and spatial frequency rather than discrete cues (number of objects) to solve the task. In another numerosity task, MaBouDi et al. showed that bumblebees (*Bombus terrestris*) did not determine the number of items by using a rapid assessment of number (as mammals do in “subitizing”) [21]. Instead, bees used a sequential enumeration strategy even when items were presented simultaneously and in small quantities. For clarity, this is not the only mechanism present in invertebrates. Recent studies have reported how flies (*Drosophila melanogaster*) are able to process visual stimuli similarly to the way primates do [22].

**Figure 1:**
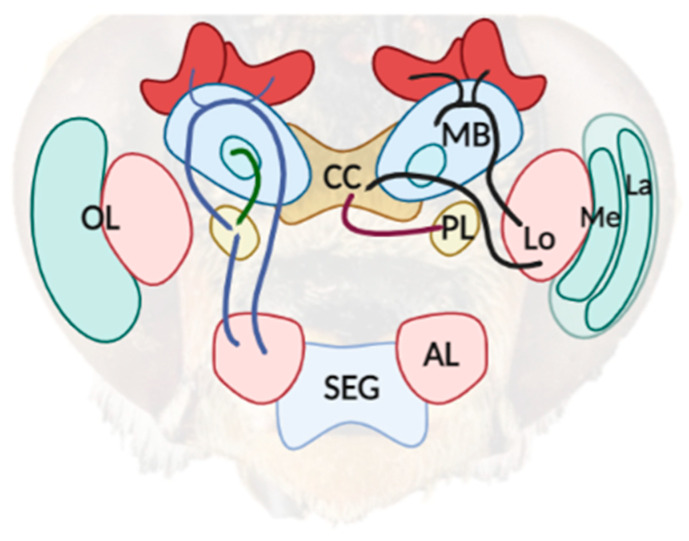
A simplified diagram of the neuroarchitecture of a bee’s brain. The sensory information captured from the compound eye is processed in the optic lobe (OL), which is comprised of the lamina (La), medulla (Me), and lobula (Lo). Two pathways (dark line) can be outlined from the posterior part of the Lo, which project axons to the mushroom body (MB) and to the central complex (CC). The lateral protocerebrum (LP) is involved in the integration of information from several areas in the bee’s brain, including visual information from the CC (purple line) and olfactory information previously processed from the antenna lobes (LB) and MB (blue line). The scheme was created with BioRender.com (accessed on 20 April 2022).

Once acquired, visual information must be integrated by high-order brain regions. Bees and several other arthropods possess a single dorsal-anterior neuronal ganglion positioned above the pharynx which is organized with defined front, middle, and back components, each specialized to process and integrate various sensory information [23]. The anterior component, called the “protocerebrum”, receives the innervation of visual organs (Figure 1). The knowledge gathered on the processing and integration of sensory information has focused on odors, mostly in honeybees (*A. mellifera*), using the proboscis extension response conditioning protocol, where the test subject is in a tube so that only the antennae and proboscis are free to move. In contrast, to study the processing of visual information, it is necessary for the individual to be in motion, limiting the study of brain activity during the task (but see [24]). Therefore, bee brains, as well as many other arthropod brains, can be divided into separate neuropil areas (zones of dense synaptic networks of neuronal processes) (for a detail reference system see [25,26]). Visual information is specifically acquired by the types of photoreceptors sensitive to different wavelengths of the spectrum [27,28,29]. The dorsal area of protocerebrum receives terminal extension originating in the optic lobe, that is, a large and complex neuropil areas involved in the analyses of visual input. Indeed, neurophysiological signals are propagated from the projections of retina photoreceptors into the first, most peripheral area of the optic lobe, the lamina [30]. This first region is composed of thousands of axon bundles derived from the ommatidia as well as neural projections of different types of monopolar cells. Axons from the lamina proceed to the inner high-order area, the medulla. Projections from the posterior part of the medulla decussate in the chiasma before innervating the inner area of the lobula [31]. Finally, information from the lobula is conveyed to different brain regions. In particular, the central complex, a specific region of the insect brain positioned in the center of the protocerebrum, comprises four unpaired neuropils [32,33]. Its primary function is to process and integrate visual information from the retina to provide an appropriate motor response [34]. The mushroom body (corpora pedunculata) has long been considered to be the principal neuropil area involved in olfactory learning and memory by receiving sensory information from the antennae [35]. Recent studies have suggested that the division between the olfactory pathways (mushroom body) and the visual pathways (central complex) was not distinct, but that both regions were crucial for learning visual and odour information in a binary choice task [36]. Although the number of neurons is smaller in bees than in vertebrates, the visual pathways in the brains of bees are still far from being fully understood [37].

## 3. Do Bees Experience Visual Illusions?

Although the study of optical illusions has always fascinated researchers of visual perception and the public, researchers have only recently begun to systematically study such phenomena outside the human sphere for the purpose of increasing our knowledge on similarities and differences in perceptual mechanisms across species [7]. Indeed, an inclusive literature search of PubMed database was carried out for articles contained the terms “visual illusion” and “invertebrate” published up to December 2021. The search yielded 52 results, including 11 studies on arthropods, 2 reviews, 2 studies on other type of sensory illusion, while the remaining were not related to the subject of interest. Despite the limited quantity of information, several studies on perception in Drosophila and other invertebrate models have used stimuli that could be classified as illusory, although this was not explicitly stated. For example, different conditioning procedures that operate in Drosophila involved exposure to patterns of vertical lines in motion [38]. In 1979, Srinivasan and Dvorak specifically studied the waterfall illusion, which consisted in the apparent motion of a static object generated by a prolonged exposure to moving patterns [39]. The authors found that the common green bottle fly (*Lucilia sericata*) exhibited a behavior similar to that observed in humans and expected from the waterfall illusion. The behavioral response appears to be elicited by the response of direction-selective neurons in the lobula.

We have already discussed how the visual perception system converts a two-dimensional image acquired through the retina into a three-dimensional scene. One of the transformations that the system uses is the identification of edges among the different elements that make up the scene. Physical characteristics of the elements such as luminance, texture, and chrominance define the contours. The system, however, “adds” contours even in the absence of real discontinuity [40,41]. These contours generate a class of phenomena called illusory contours (Table 1).

In the last 30 years, contour illusions have proven to be fundamental for the development of models that explain how sensory information is processed and integrated within the visual system. In two preliminary studies, Van Hateren et al. and Horridge et al. extended the studies on the perception of illusory contours in honeybees [42,43]. In both studies, the researchers trained individual bees to discriminate patterns of vertical and horizontal lines and, subsequently, stimuli of subjective illusory contours. Bees were also presented with Kanizsa-type rectangles, a class of illusory contours generated by the spatial arrangement between elements with high-contrast borders [48]. When trained with regular line patterns, bees showed no preference for boundary illusion stimuli. Conversely, when bees were trained with non-regular line patterns and which simulate boundary illusions, they showed a preference for illusory solids (i.e., solids generated by the boundary illusion) with the same orientation as the patterns they were trained to.

Another interesting class of illusory phenomena is color illusions (Table 1). “Color” is a property of an object defined by the mind. Indeed, color is an electromagnetic phenomenon generated from the reflection of light on the object. The reflected light stimulates the retina and the brain processes the visual information, enabling color perception. As a result, color is an illusion per se. One classical color illusion is Mach bands, defined after the phenomenon was discovered by the physicist Ernst Mach [49]. It results from the contrast between the edges of grey gradients of relatively close intensity in a square-wave grating. The alternating color pattern increases sensory perception of the luminance channel in the retina by inhibiting the spreading of action potentials, a motor neural response known as lateral inhibition. Several color illusions stem from alternating patches of color with different luminance/brightness. The Fechner color effect is induced by a rapidly alternating moving pattern of black and white stimuli. Although bees possess trichromatic color vision similar to that of the human visual system, their visible spectrum is shifted toward shorter wavelengths, encompassing the range from UV to green [50]. When presented with the Benham pattern, a certain black-and-white concentric circle pattern, honeybees showed similar behaviors to those expressed by humans in the presence of the illusory stimulus [44]. The Craik–O’Brien–Cornsweet illusion, or simply the Cornsweet illusion, is another optical color illusion, but it differs from the described Mach bands or Fechner phenomena. In the Cornsweet illusion, regions close to the lighter part of the edges are perceived as lighter, while regions close to the darker area are perceived as darker. Davey et al. investigated whether honeybees experienced the Cornsweet illusion [45]. Bees were trained to discriminate between square-wave gratings differing in the luminance contrasts between close elements. Again, bees showed behavioral similarities to humans when faced with the illusory pattern. A possible explanation proposed by Davey et al. relied on the antagonism interaction between the center and surround regions of the receptive field of photoreceptor cells.

The described studies concern phenomena that affect the visual pathways of image acquisition. An interesting phenomenon first proposed by Navon concerns the differing amount of attentional resources deployed for some aspects of an image [51]. In a seminal study, Navon pointed out that subjects responded faster when discriminating between global versus local features of a scene. In his work, precisely described as “Forest before trees”, Navon emphasizes how humans paid more attention to the global information of the scene than to the individual elements that made it up. In particular, the global possesses unique characteristics not presented at the level of individual elements. For example, density is not visible at the level of the single element, but it emerges when we consider the various elements as a group (Table 1). Humans and other vertebrates generally use global processing for perceiving the world, that is, the tendency to process the overall images of a scene rather than a collection of the separate features which form it [52,53,54]; (but see [55,56]). In a few studies, Avarguès-Weber et al. investigated whether honeybees prioritize global or local information by setting a scene with these two levels in competition [15,46]. Even when local information was accessible, honeybees first relied on the global information, that is, the spatial arrangement of an entire scene, to perform visual discrimination, suggesting that prioritizing of global processing could be a spontaneous mechanism for analyzing a complex visual scene in bees in the same way as in humans. Indeed, global processing might be more resilient to an inconsistent visual scene for moving animals, which acquire information from different viewpoints [57,58]. Another well-known example of global information effect comes from the Ebbinghaus or Titchener circle illusion, i.e., two identical targets are perceived differently when surrounded by small/large or close/distant external inducers. Howard et al. asked the question of whether honeybees are affected by contextual size illusions [47]. Honeybees were trained to discriminate between stimuli differing in size, and then tested with a contextual size illusion. Honeybees showed a preference similar to those experienced by humans. However, size perception was influenced by the conditioning procedure: in a restricted viewing condition, honeybees showed no preference for either one of the two illusory stimuli.

## 4. The Neural Root of Illusory Misperception

An interesting question that has long fascinated neuroscientists and behavioral psychologists is whether and how nonhuman species perceive visual illusions. The possibility of comparing the behavioral responses of different species when faced with illusory stimuli allows us to understand how different perceptual systems have evolved to overcome similar ecological pressures. Among the most studied nonhuman species, primates have played a central role in understanding the mechanisms underlying visual perception. The primary visual cortex (V1) is a specific region of the cerebral cortex that receives sensory input from the lateral geniculate nucleus. This area is highly specialized for pattern recognition by transforming visual inputs into neural firing rates. Then, neurophysiological information is transmitted through two primary pathways known as the ventral and dorsal streams. The first stream is critical for visual perception, while the dorsal stream mediates visual control of moving actions and the location of objects in the environment. Due to its involvement in visual processing, the ventral stream might be the target area in which the sensory mechanism is affected by the illusory scene. For example, the brightness illusion concerns the misperception of equal objects due to the apparent contrast of the object to the background on which it is placed. The misperception event might manifest during the early pathways of sensory input processing in luminance-responsive cells in color-activated regions of V1 and in the secondary visual cortex in macaque monkeys (*Macaca fuscata*) [59,60]. These regions encode the response to the physical modulation of luminance due to the presence of neurons with restricted receptive fields. Thus, the misperception of the brightness illusion and other illusory phenomena, for example, illusory contours [61], might be ascribed to a limited and easy feedforward mechanism of processing of sensory inputs [62].

This scenario becomes even more complicated when we consider bees. Although bees exhibit behavior similar to that of humans in response to the perception of illusory stimuli, they do not possess a cortex. Consequently, the cortex present in mammalian brains is not a necessary condition for triggering the misperception. It is possible that misperception occurs at an early level of the visual pathways. Regarding the visual pathways in insects, the photoreceptors transform visual inputs from the eye to neurophysiological signals to the optic lobe. The spatial arrangements of such neural ganglia, especially in the inner region of the lamina, remain constant throughout the different layers of the optic lobe, thus, providing a retinotopic organization of those neurons involved within the visual pathways. The chiasma, which connects the posterior region of the medulla and innervates the lobula, might potentially play a role in processing complex visual information [63]. A recent work by Agrochao et al. showed how the perception of an illusion of movement in *Drosophila* was generated by unbalanced contributions of distant-selective neurons’ responses in stationary edge sampling [64]. Flies whose T4 and T5 motion neurons (detection-selective neurons that respond exclusively to dark edges or moving light presented in the second chiasma) had been ablated did not experience the illusion of movement. It is interesting to note that the same mechanism has also identified in humans [63]. Indeed, the pathway for visual information in vertebrates is through the retina and lateral geniculate nucleus to the primary visual cortex. The transition from sensory stimulation of photoreceptors to electrochemical signals leading to a behavioral response is subject to the different properties of the areas passed along the way [65,66,67].

## 5. Discussion

The information collected in this review suggests that bees show a human-bias judgement when presented with stimuli made for humans. Can we, therefore, claim that bees perceive illusions? According to the current literature, bees do perceive illusions, but not necessarily in the same way as humans.

Researchers usually propose theories and subsequently conduct experiments to support them. Unfortunately, this approach has limitations when we are interested in understanding cognitive abilities in nonhuman species. Indeed, animals can learn different strategies to solve a task, but the outcome is similar to what we expect. Let us take as an example the color illusion presented by Davey et al. [45]. Color illusions such as Mach bands originate from overstimulation of the sensory neurons of the human retina. Recent work in flies seems to confirm that sensory neurons present in the early stage of visual pathways increased their activity when exposed to square-wave patterns [68]. It might also be possible that bees learn to discriminate stimuli based on the perceived hue generated from the spatial arrangement of elements and/or their sampling behavior. Indeed, Srinivasan et al. found that the perception of color by bees was affected by previous exposure to a rotated square-wave pattern [44]. Even though the behavioral outcome is the same, bees can learn alternative strategies for making their decision, depending on the training protocol employed or on experience previous to the stimuli [69,70,71]. Considering multiple features of animal behavior (e.g., decision time and the pattern of movement) and not only their simple choice would certainly strengthen the comprehension of the strategies that animals undertake before making a decision.

As Avarguès-Weber et al. clearly pointed out [15,46], the methodology adopted for investigating our hypotheses plays an important role in the outcome. Many of the stimuli adopted to verify whether nonhuman species perceive visual illusions are based on human perception. Several procedures employ monitors to present controlled visual stimuli. The problem is that screens have a filter that transmits human-visible light, which can affect the perception of stimuli in other species. It is common to train an insect by presenting stimuli with a relatively large stimulus to individual’s size ratio as compared with ones used for humans and nonhuman primates. Generally, human subjects are presented with relatively small stimuli (~2–10 cm) as compared with their size, while insects have to discriminate between stimuli two or more times larger than their own size. Although the basic neural mechanisms for the acquisition of visual information seem common among species, we cannot affirm that different species use the same visual principles for making decisions. For example, bees perceive ultraviolet (UV) light, but humans do not. Many flower species have UV-absorbing areas or specific-colored areas that may make them look bigger or otherwise more attractive to pollinators. It is advantageous for flower species to be able to influence how pollinators perceive their attractiveness. Indeed, the salience of flowers’ characteristics reflect natural selection based on pollinator preferences [72]. Methodological differences between species, and even within the same species, can influence the robustness of comparative results. As already mentioned in the introduction of this review, species have developed various systems for perceiving the external world, and such systems might not be following our expected hypothesis. Further studies should be careful about the ecology of species when planning to perform comparative experiments.

In conclusion, do bees experience visual illusion? The current literature suggests that they do, but the mechanisms underlying such phenomena remain poorly understood. Visual illusions provide windows into the mechanism underlying the visual system [4]. Despite this, we are still far from understanding the causes that generate the illusory phenomena. Undoubtedly, further studies are needed to understand the evolutionary origin of vision systems across species. We have reported how bees [15,73], and other social hymenoptera (such as wasps) [74,75,76], are able to categorize and recognize the features of faces. More interesting is the potential role of the environment in influencing the development of such cognitive functions among species and even within the same species can be influenced by social and physical environment. For example, socially isolated wasps of the species *Polistes fuscatus* do not develop face recognition, especially when deprived of social signals in the early stage of life [77]. Moreover, geographical distance has been shown to increase phenotype variability in the capacity for face recognition in two *P. fuscatus* populations [78]. Species with and without face recognition capacity possess different growth rates and complexities of neural connectivity in their anterior optic tubercle, suggesting a possible neural area which mediates such cognitive capacity [79]. By manipulating the individual experience of environment, we might be able to influence neural and cognitive development and, consequently, understand which areas are involved in the misperception of illusory stimuli [80]. The rapid generation time and short lifespan, the compact genome size, the well-organized brain architecture, as well as other advantages, suggest invertebrates could be a successful model for investigating the genetic and neural components of behaviors. Nevertheless, all the parallelisms emerging from this review might reflect the fact that humans, nonhuman vertebrates, and certain invertebrates, despite the markedly different architecture of their eyes, have evolved similar processing mechanisms to deal with similar environmental pressures [7].

## Figures and Tables

**Table 1:** Diagram and descriptions of illusory phenomena similarly experienced by bees and human.

Class of Illusion	Description	Illusory Stimulus
Illusory contours	For humans, a white rectangle is generated from the identification of an edge between the different ”Pac-Man” elements that create the scene. Bees showed similar susceptibility of those expressed by humans when presented with stimuli with high-contrast borders [42,43].	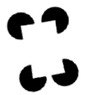
Color Illusion	This class of illusion is generated from the contrasts between the pattern generated from moving elements (the Fechner color illusion) [44] or physical similarities (i.e., brightness and luminance) between elements (Cornsweet illusion) [45]. Humans and bees show behavioral similarities when presented with high-colored contrast stimuli.	The Fechner color 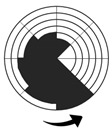
		The Craik–O’Brien–Cornsweet Illusion (Cornsweet Illusion) 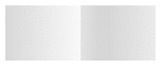
Global Perception	The tendency to process the overall scene rather than a whole set of single elements which define it seems widespread from humans to bees [15,46].	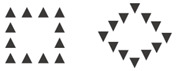
Contextual Illusion	Humans and bees are susceptible to the contextual illusion. The sizes of two identical squares are misperceived due to the background that surrounds each of them [47].	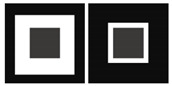

## Data Availability

Not applicable.
